# Interventions in Primary Care and their contributions to improving equity in health

**DOI:** 10.1186/s12939-015-0284-6

**Published:** 2015-12-14

**Authors:** Ana Lorena Ruano, John Furler, Leiyu Shi

**Affiliations:** Centro de Estudios para la Gobernanza en los Sistemas de Salud (CEGSS) Guatemala and Center for International Health, University of Bergen, Bergen, Norway; Department of General Practice| Faculty of Medicine, Dentistry and Health Sciences University of Melbourne, Melbourne, USA; The Primary Care Policy Center Department of Health Policy and Management, John Hopkins Bloomberg School of Public Health, Bloomberg, USA

Primary Care constitutes the first and most important point of contact between the health system and the population it is there to serve. Through it, essential, continuous, comprehensive, and coordinated care that does not discern between gender, disease or geographical location can be provided [1]. Whether delivered by physicians or other members of the primary care workforce, evidence shows that strong primary care is associated with better access to and quality of care, and with better health outcomes. Evidence also suggests that interventions can specifically strengthen primary care to improve access and that in turn improves equity of outcomes [2].

Today, the International Journal for Equity in Health announces the launch of a new thematic series that looks at the contributions that primary care interventions have on equity levels in health systems. Our goal is to provide a space to document and analyze the primary care reforms and interventions that have been undertaken in recent years by communities, organizations, countries and world agencies. While the thematic series will continue in a rolling fashion, today we publish the first set of papers in the series that focuses on research on successful stories, as well as lessons learned so that previous experiences may inform future efforts that can be implemented at the global, national or community level.

There have been several primary care interventions that aim to improve equity levels in China and Tibet, and this first article collection reflects this with four articles. First, we look at usual source of care and its relationship with primary care in Guandong province [3]. This study, which is the first to compare primary care contributions through controlling for the source of care, found that patients that had a usual primary care provider reported higher quality of medical care experiences. In line with these findings, another study also carried out in Guangdong examined four models of primary care delivery models for people with chronic conditions and found that an insurance mandate that uses family practice physicians as ‘gatekeepers’ seemed to work best when it came to promoting access and quality care for patients [4]. The third paper in our thematic series looks at integrated care delivery and health care seeking by people with chronic conditions in Henan province and found the integrated care delivery model was critical in guiding patient’s health seeking behavior, reduced health inequities, and mitigated disparities for older patients [5]. Our fourth paper evaluates the contribution that primary care has to health in Tibet, and found a positive association between higher quality primary care and better self-rated health status. The study findings point to the importance of strengthening this level of care during future health system reforms [6].

From Tibet we move to Sweden, where primary care is seen as the key to developing equitable health care. The study found that patients/clients perceived that their ethnic origin and mental health status played an important role in the quality of care that they received, and that employing health providers that are proficient in the languages that are spoken in the communities they serve is a way of providing more equitable primary care services [7]. In Manitoba, a study found that pay-for-performance for primary care physicians had a limited impact on vaccination rates and in this study did not appear to reduce health inequity [8]. To close this collection, the article by Carriel et al. looks at the integration of traditional indigenous medicine into primary care in Nicaragua, and found that careful study and monitoring of legislation is needed in order to implement and carry out policy alignments that can lead to the full integration of his in health plans at the local and national levels.

The International Journal for Equity in Health welcomes further contributions to this rolling thematic series, particularly as countries around the world continue to grapple with issues around reforming and strengthening primary care systems in all settings.
